# Comparative analysis of morphological traits and photosynthetic parameters as well as carbon accumulation characteristics of six typical shrub species in the Qilian Mountains

**DOI:** 10.3389/fpls.2025.1729429

**Published:** 2026-01-22

**Authors:** Rong Zhou, Na Wei, Yaoyao Shangguan, Hu Zhao, Bin Chen, Yin Miao, Hongmei Liu, Xiaobin Xie, Gang Chen, Jingzhong Zhao, Dong Lv

**Affiliations:** 1 Academy of Water Resource Conservation Forests of Qilian Mountains in Gansu Province, Zhangye, China; 2 College of Forestry, Gansu Agricultural University, Lanzhou, China

**Keywords:** arid zone shrubs, biomass allocation, functional traits, photosynthetic characteristics, whole-plant carbon storage

## Abstract

**Introduction:**

Shrubs are key components of arid ecosystems, and their functional traits directly influence ecological adaptability and productivity. Current research pays insufficient attention to the synergistic relationship between the overall morphological structure and leaf physiological functions of shrubs. This study focused on six typical shrub species in the arid zone of the Qilian Mountains, aiming to analyze interspecific differences in functional strategies from a “morphology–photosynthesis” synergy perspective.

**Methods:**

We selected six typical shrub species (e.g., *Cotoneaster multiflorus, Prunus pedunculata, Caragana arborescens*, and *Lonicera rupicola*) and comprehensively measured their morphological traits (plant height, basal diameter, root length, biomass allocation, etc.) and photosynthetic physiological parameters (net photosynthetic rate, transpiration rate, stomatal conductance, etc.).

**Results:**

The results showed that: (1) Morphologically, *C. arborescens* exhibited significantly greater plant height (205.17 cm) and whole-plant dry weight (303.03 g), while *L. rupicola* had deeper root systems (>40 cm); (2) Photosynthetically, the diurnal net photosynthetic rate displayed unimodal and bimodal patterns, primarily driven by photosynthetically active radiation, with *L. rupicola* and *C. arborescens* showing the highest estimated daily leaf-level carbon assimilation potential (6.93 and 5.86 g·m⁻²·d⁻¹, respectively); (3) A “scale decoupling” existed between whole-plant carbon storage capacity and leaf-level carbon assimilation potential: *C. arborescens* had the highest whole-plant carbon storage (120.86 g/plant) but not the highest per-unit-leaf-area assimilation efficiency, whereas *L. rupicola* exhibited high leaf-level efficiency but moderate whole-plant storage; (4) The six shrubs were classified into three strategic types based on biomass allocation and carbon storage: *C. arborescens* as “high-accumulation, stem-dominated”; *C. multiflorus, Lonicera ferdinandi, P. pedunculata, L. rupicola* as “balanced investment”; *Euonymus phellomanus* as “conservative, belowground-investment”.

**Discussion:**

By integrating leaf-scale carbon assimilation potential estimates with whole-plant carbon storage measurements, this study systematically revealed the scale-decoupling phenomenon and established a more rigorous framework for assessing shrub carbon sinks. The findings demonstrate significant diversity in the synergistic differentiation of morphological and photosynthetic traits as well as carbon accumulation strategies among arid-zone shrubs. Vegetation restoration should select corresponding functional species based on objectives such as rapid carbon accumulation or stress adaptation, providing theoretical support and practical guidance for ecological restoration and carbon sink enhancement in arid regions.

## 1 Introduction

Under the backdrop of global climate change, enhancing the carbon sink capacity of terrestrial ecosystems has become a critical pathway to mitigate rising atmospheric CO_2_ concentrations (Sawirdin et al., 2023). As a significant component of terrestrial carbon pools, the carbon sink function of arid and semi-arid ecosystems profoundly influences the global carbon balance (Sun et al., 2024). Shrubs, as key functional components of such ecosystems, play an irreplaceable role in maintaining regional carbon balance and ecological security due to their remarkable environmental adaptability, drought tolerance, and potential carbon sequestration capacity (Gao et al., 2023). This is particularly evident in the arid regions of northwest China, where shrubs contribute significantly to soil and water conservation, windbreak and sand fixation, and carbon sequestration (Li et al., 2023). However, current research remains predominantly focused on tree-dominated systems (Zhou et al., 2021; Wang et al., 2024a), with insufficient attention paid to shrubs in arid areas. Moreover, significant “leaf-scale limitations” persist in assessing their carbon sink functions—specifically, an overreliance on per-unit-leaf-area photosynthetic parameters, while often overlooking the systematic contributions of whole-plant morphological structure and biomass allocation (Yan et al., 2023; Wang et al., 2023a).

Biomass, as the direct carrier of carbon storage, reflects plants’ growth adaptation strategies through its above- and below-ground allocation patterns (Siddique et al., 2024), and is also key to understanding ecosystem carbon partitioning and storage. Research has shown that relying solely on leaf-scale photosynthetic estimates is insufficient to fully capture a plant’s actual carbon accumulation capacity, because different species exhibit significant variations in traits such as leaf area index and crown morphology. Therefore, it is necessary to integrate morphological characteristics (plant height, basal diameter, and biomass allocation) with photosynthetic physiological parameters, in order to systematically compare the relationship between leaf-level carbon assimilation potential and whole-plant carbon storage characteristics (Xu and Trugman, 2021).

In recent years, trait-based and photosynthetic physiological studies on plant adaptation have offered new perspectives for deepening the understanding of shrub carbon sequestration mechanisms. Research has demonstrated that plants can develop coordinated adaptive strategies through their morphological, anatomical, and physiological traits in response to environmental stress (Pan et al., 2024; Singh, 2024). For instance, under combined drought and high-temperature stress, grapevine (*Vitis vinifera*) leaves exhibit significant developmental stage-dependent variations in leaf structure and metabolite accumulation (Kolyva et al., 2025). Furthermore, the dynamic degradation mechanism of calcium oxalate crystals in leaves can maintain basal carbon assimilation through “alarm photosynthesis” under stomatal limitation, highlighting the close association between structural traits and carbon acquisition function (Kolyva et al., 2023). Meanwhile, studies on the physiological adaptation mechanisms of understory shrubs to climate change have revealed significant interspecific differences in the regulation of photosynthetic optimum temperature and responses in stomatal conductance among different shrub species, with soil moisture emerging as a key environmental driver influencing their physiological responses (Carter et al., 2020).

The Qilian Mountains serve as a critical ecological security barrier in the arid and semi-arid regions of Northwest China, providing unique hydrological regulation and carbon sink functions (Zhao et al., 2025; Shangguan et al., 2025). However, this region also faces multiple stressors, including water scarcity, poor soil fertility, and vegetation degradation (Yin et al., 2023). Against this background, this study focuses on six typical shrub species in the arid zone of the Qilian Mountains. By systematically measuring their morphological traits (plant height, basal diameter, root system architecture, biomass allocation, etc.), photosynthetic physiological parameters (net photosynthetic rate, transpiration efficiency, stomatal conductance, etc.), and carbon storage across different organs, we aim to analyze functional strategy variations among species from a multi-scale perspective that integrates morphology, photosynthesis, and carbon storage.

The objectives of this study are: (1) to systematically compare morphological differences among the six shrub species and analyze diurnal variation patterns of photosynthetic parameters and their responses to environmental factors; (2) to reveal the contribution of biomass allocation and organ-specific carbon storage patterns to whole-plant carbon accumulation capacity; and (3) to systematically compare the differences and connections between leaf-scale carbon assimilation potential estimated from photosynthetic parameters and whole-plant carbon storage characteristics obtained through destructive harvesting.

It should be explicitly noted that the “carbon assimilation potential” estimated from photosynthetic parameters during the peak growing season in this study is only an instantaneous physiological indicator at the leaf scale, reflecting the upper limit of potential carbon acquisition under ideal conditions. It does not account for respiratory carbon losses, carbon partitioning, phenological changes, or carbon fluxes during the non-growing season. Therefore, this metric should not be directly equated with actual carbon sink capacity at the plant or ecosystem scale. The results presented here are intended solely for comparing interspecific differences in leaf-scale carbon-acquisition efficiency and their association with whole-plant carbon storage performance. Future research should incorporate continuous cross-seasonal and multi-year monitoring to establish more comprehensive photosynthesis-environment response models, enabling more accurate assessment of shrub carbon sink functionality. This study aims to provide multi-scale trait-based evidence for understanding the ecological functions of arid-zone shrubs and for species selection in vegetation restoration efforts.

## 2 Materials and methods

### 2.1 Study area description

This study was conducted at the Longqu National *Picea crassifolia* Seed Orchard (100°13′42″ E, 38°48′41″ N) in Zhangye City, Gansu Province, a region characterized by a typical continental desert climate with a mean annual temperature of 6.8°C, annual precipitation below 200 mm (showing uneven seasonal distribution), and annual evaporation reaching 1653.0 mm (8.5 times the precipitation), along with a frost-free period of 152 days. The predominant soil types in the area are sierozem, aeolian sandy soil, and brown calcic soil, which exhibit loose texture, low organic matter content (<1%), alkaline pH (7.5-8.5), and poor water retention capacity. The region is subject to year-round arid stress dominated by evaporation, with extremely scarce water resources. These harsh climate, soil, and hydrological characteristics provide an ideal setting for studying the adaptive strategies and carbon sink functions of shrubs under extreme arid conditions, while also posing significant challenges to vegetation restoration in the region.

### 2.2 Study subjects

This study selected six representative shrub species that have been successfully introduced and acclimatized at the Longqu National *Picea crassifolia* Seed Orchard, demonstrating high environmental adaptability and survival rates, including *Cotoneaster multiflorus*, *Prunus pedunculata*, *Lonicera ferdinandi*, *Lonicera rupicola*, *Caragana arborescens*, and *Euonymus phellomanus*, all of which were healthy individuals without visible signs of disease or pest damage. Using Randomized Block Design with five-year-old seedlings (spring 2019 cohort) to ensure genetic consistency (Shangguan et al., 2025), each species was planted in separate plots containing four rows of 20 plants each with three replicates, maintaining uniform spacing of 2×3 m between plants to control for planting density effects on experimental outcomes.

### 2.3 Measurement indicators and methods

#### 2.3.1 Morphological index measurement

In this study, 30 individual plants per species were randomly selected as samples for morphological measurements. This sample size was determined to balance statistical reliability with the practical workload required for precise, plant-by-plant measurement of traits such as basal diameter, plant height, and crown width, given the substantial morphological variation among shrubs. Basal diameter was measured with a digital caliper with a precision of 0.01 mm. Plant height (vertical distance from ground level to the apex of the shrub) and crown width (calculated as the average of the maximum horizontal diameters measured in the north-south and east-west directions) were measured using a 5 m measuring tape. All morphological measurements were conducted under standardized meteorological conditions to minimize environmental interference and ensure data accuracy and comparability. To determine root length, the soil around the root system was carefully excavated to fully expose the roots. The length of the deepest lateral root was then measured directly using a 5 m tape; this parameter serves as an indicator of the plant’s ability to access deep soil water resources (Yao et al., 2014).

#### 2.3.2 Biomass index measurement

In this study, the whole-plant harvesting method was employed to determine shrub biomass (Xu et al., 2014). The specific procedures were as follows: For each shrub species, five individual plants were selected as measurement samples. With the shrub main stem as the center, soil within a radius of 1.5 m was carefully removed in layers to preserve the integrity of the root system as much as possible. The harvested plants were then separated into roots, leaves, and branches (stems), and the fresh weight of each organ was measured using an electronic balance with an accuracy of 0.01 g. Data were recorded promptly to minimize moisture loss. The separated organs were placed in an oven at 105°C for 2 hours for enzyme deactivation to prevent the decomposition of organic matter (Semenov et al., 2022). Subsequently, the oven temperature was adjusted to 85°C, and samples were dried to constant weight, defined as when the difference between two consecutive weight measurements was less than 0.01 g. The dry weight of each organ was accurately weighed and recorded for subsequent biomass analysis. The moisture content (%) was calculated using the following formula:

(1) $$\text{Moisture Content}=\frac{\text{Fresh Mass }-\text{ Dry Mass}}{\text{Dry Mass}}$$

#### 2.3.3 Leaf trait measurements

Specific Leaf Area (SLA) Determination: To accurately assess leaf traits, three representative standard plants of each species (Utkin et al., 2022) were selected, from which 50–100 fully expanded, undamaged mature leaves were randomly collected from the four cardinal directions (east, west, south, north) of the canopy (Garnier et al., 2001). The harvested leaves were smoothly affixed onto transparent adhesive tape with 100% light transmittance, and their area was precisely measured using an Li-3000C leaf area meter (LI-COR, USA), with raw data meticulously recorded; to enhance measurement accuracy, each leaf was measured three times and the average value was taken as the final leaf area. Following leaf area measurement, the leaves underwent a 2-hour enzyme deactivation treatment at 105°C to halt enzymatic activity, after which the temperature was reduced to 85°C for drying until constant weight was achieved (defined as weight changes of less than 0.001 g), whereupon the dry weight was measured using an electronic balance with 0.001 g precision, with the resulting data used for specific leaf area analysis according to the formula:

(2) $$\text{SLA}=\frac{\text{S}}{{\text{W}}_{dry}}$$

In Equation 2, SLA represents the specific leaf area (cm²·g^-^¹), S denotes the leaf projected area (cm²), and ${\text{W}}_{dry}$ indicates the leaf dry weight (g).

The Leaf Area Index (LAI) was calculated using the following formula:

(3) $$\text{LAI}=\frac{\text{W}\times \text{SLA}}{\text{C}}$$

In Equation 3, LAI represents the leaf area index, W denotes the total leaf dry weight per plant (kg), and C indicates the crown projection area (m²).

#### 2.3.4 Photosynthesis parameter measurements

The measurement of photosynthetic parameters in this study was conducted during summer when plant growth metabolism is most vigorous and photosynthetic capacity is highest (Dong et al., 2024). Measurements were performed using a Li-6800 portable photosynthesis system (LI-COR Biosciences, USA) from August 22 to 29, 2024. Under clear, cloudless, and windless or breezy natural conditions, the net photosynthetic rate of plants was measured every two hours between 8:00 and 18:00 daily to capture diurnal photosynthetic dynamics (Zhang et al., 2023). Photosynthetic measurements strictly followed standard procedures to maintain a stable leaf chamber environment. To ensure stable and homogeneous gas concentrations during measurement, an external buffer bottle was used to minimize airflow fluctuations. The buffer bottle was positioned upwind of the measurement point, away from human activity, and approximately 1 meter above ground level to sample representative ambient air.

Specific procedures were implemented as follows: three healthy, disease-free standard plants per shrub species were selected, with sun-exposed mature leaves of consistent size and growth status chosen from each plant. Each leaf underwent three replicate measurements while ensuring complete coverage of the leaf chamber without mutual shading to maintain uniform light distribution. Five instantaneous readings were recorded per measurement, with their average calculated as the final result.

The measured photosynthetic parameters included net photosynthetic rate (Pn), transpiration rate (Tr), stomatal conductance (Gs), and intercellular CO_2_ concentration (Ci). Concurrently, the following environmental factors were recorded: photosynthetically active radiation (Qin), air temperature (Ta), leaf temperature (Ti), relative humidity (RH), and vapor pressure deficit (VPD) (Nguyen et al., 2021b). These environmental data facilitate the analysis of relationships between photosynthetic physiological parameters and ambient conditions. Water use efficiency was calculated using the formula:

(4) $$\text{WUE}=\frac{\text{Pn}}{\text{Tr}}$$

In Equation 4, WUE represents water use efficiency, Pn denotes the net photosynthetic rate, and Tr indicates the transpiration rate.

The stomatal limitation value was calculated using the formula:

(5) $${\text{L}}_{s}=1-\frac{{\text{C}}_{i}}{{\text{C}}_{a}}$$

In Equation 5, Ls represents the stomatal limitation value, Ci denotes the intercellular CO_2_ concentration, and Ca indicates the ambient atmospheric CO_2_ concentration.

#### 2.3.5 Carbon sequestration and oxygen release measurements

The daily net assimilation per unit leaf area is represented by the area enclosed between the diurnal net photosynthetic rate curve and the time axis in a net photosynthetic rate diurnal variation graph, and can be calculated using a simple integration method (Schaaf et al., 2011; Li et al., 2010). The formula for daily net assimilation is as follows:

(6) $$P=\sum_{i=1}^{j}\frac{P_{i+1}+P_{i}}{2}\times (t_{i+1}-t_{i})\times \frac{3600}{1000}$$

In Equation 6, P represents the daily assimilation per unit leaf area (mmol·m^-^²·d^-^¹); P_i_ denotes the instantaneous photosynthetic rate at the initial measurement point (μmol·m^-^²·s^-^¹); P_i+1_ indicates the instantaneous photosynthetic rate at the subsequent measurement point (μmol·m^-^²·s^-^¹); t refers to the instantaneous time at the initial measurement point (h); t_i+1_ corresponds to the instantaneous time at the subsequent measurement point (h); and j signifies the number of measurement intervals.

The estimation of nighttime dark respiration consumption refers to relevant research methods on typical shrubs in the arid and semi-arid regions of northwest China, and is accounted for as 20% of the daytime net assimilation. This ratio has been validated and applied in studies on the carbon sequestration and oxygen release capacities of typical shrubs in the Helan Mountains (such as *Picea crassifolia*, *Berberis thunbergii*, *Lonicera microphylla*, etc.), and is suitable for calculating related parameters in similar arid and semi-arid habitats (Chen et al., 2021).

The formula for converting the daily CO_2_ fixation per unit leaf area (WCO_2_, unit: g·m^-^²·d^-^¹) on the measurement day is as follows:

(7) $${\text{W}}_{CO_{2}}=\frac{\text{P}\times \left(1-0.2\right)\times 44}{100}$$

According to the method described by Chen et al. (2021), the daily oxygen release per unit leaf area (WO_2_, unit: g·m^-^²·d^-^¹) is calculated using the following formula:

(8) $${\text{W}}_{O_{2}}=\frac{\text{P}\times \left(1-0.2\right)\times 32}{100}$$

#### 2.3.6 Determination of carbon content in shrub organs

After measuring the dry biomass of each organ, the samples were ground into a fine powder using a plant grinder (sieve diameter: 0.5 mm) following drying. From each organ, 5 g of powdered sample was sealed and stored in a desiccator protected from light for subsequent carbon (C) content analysis. Plant organic carbon content was determined using the potassium dichromate-sulfuric acid oxidation method (Yu et al., 2024). Each sample was analyzed in triplicate, and the mean value was taken as the carbon content of the sample.

The whole-plant weighted average carbon content was calculated based on the biomass proportion of each organ using the following formula:

(9) $$C_{avg}=\frac{\sum M_{i}R_{i}}{M_{i}}$$

In Equation 9, ${\text{C}}_{\text{avg}}\;$ represents the whole-plant weighted average carbon content, ${\text{R}}_{\text{i}}\;$ denotes the carbon content of organ i for a given species, and $\;{\text{M}}_{\text{i }}$ is the biomass of organ i (where i=leaf, stem, root).

Carbon storage was calculated as:

(10) $$C_{t}=\sum R_{i}M_{i}$$

In Equation 10, C_t_ represents the whole-plant carbon storage, and R_i_ denotes the carbon content of organ i for a given species.

### 2.4 Data analysis

Raw data were organized using Microsoft Excel. Experimental indicators were analyzed via one-way ANOVA in SPSS 26.0, with the LSD method applied to test the significance of differences among various treatments. Cluster analysis and correlation heatmaps were performed using Origin Pro 2021.

## 3 Result and analysis

### 3.1 Daily dynamics of environmental parameters

The diurnal patterns of environmental factors were primarily driven by solar radiation. As shown in **Table 1**, Photosynthetically active radiation (Qin) and air temperature (Ta) reached their maxima at 12:00 (726.14 μmol·m^-^²·s^-^¹ and 24.54°C, respectively). This period of peak insolation was also associated with the highest vapor pressure deficit (VPD, 1.69 kPa) and the lowest relative humidity (RH, 48.76%). After 14:00, Qin declined sharply to 37% of its peak, while Ta decreased only moderately. The atmospheric CO_2_ concentration (Ca) reflected biological activity, decreasing markedly from 8:00 to 12:00 due to photosynthetic uptake, before rising to its highest value (423.03 μmol·mol^-^¹) at 18:00.

**Table 1:** Diurnal variation of environmental factors.

Time	Ambient CO_2_ concentration (Ca) (µmol·mol^-^¹)	Relative humidity (RH) (%)	Vapor pressure deficit (VPD) (kPa)	Photosynthetic photon flux density (Qin) (µmol·m^-^²·s^-^¹)	Atmospheric temperature (Ta) (°C)
8:00	415.38 ± 0.57 b	49.91 ± 0.28 b	1.1 ± 0.01 e	297.69 ± 9.03 d	19.41 ± 0.1 d
10:00	403.58 ± 0.36 c	48.81 ± 0.11 cd	1.52 ± 0.02 bc	681.17 ± 16.62 b	23.55 ± 0.12 b
12:00	400.15 ± 0.32 d	48.76 ± 0.12 d	1.69 ± 0.02 a	726.14 ± 21.69 a	24.54 ± 0.13 a
14:00	400.02 ± 0.31 d	49.37 ± 0.15 bc	1.55 ± 0.02 b	457.83 ± 18 c	23.72 ± 0.14 b
16:00	400.51 ± 0.26 d	48.91 ± 0.1 cd	1.49 ± 0.01 c	277.8 ± 6.45 d	23.55 ± 0.1 b
18:00	423.03 ± 0.73 a	51.71 ± 0.31 a	1.25 ± 0.01 d	57.28 ± 1.56 e	21.77 ± 0.13 c

Data were collected using a Li−6800 portable photosynthesis system, under clear and windless conditions from August 22 to 29, 2024. VPD denotes the vapor pressure deficit calculated based on leaf temperature; Qin represents the photosynthetically active radiation (PPFD) incident on the leaf surface. Different lowercase letters within the same column indicate statistically significant differences among sampling time points (P< 0.05, one−way ANOVA followed by Duncan’s multiple range test).

### 3.2 Characteristics of the six shrub species

The growth characteristics of the six shrub species showed significant differences (p< 0.05) (**Table 2**). *C. arborescens* had the significantly greatest plant height (205.17 cm), while the basal diameter of *L*. *ferdinandi* (27.47 mm) was greater than that of the other species. In terms of root development, *L*. *rupicola* had a significantly greater root length (42.17 cm) than the other species, whereas *L*. *ferdinandi* had the shortest root length (26.23 cm). Regarding crown width, the east-west crown spread of *C*. *arborescens*, *P. pedunculata*, and *C*. *multiflorus* (68.0 - 68.1 cm) was significantly greater than that of *E*. *phellomanus* (37.60 cm). Overall, *C*. *arborescens* exhibited advantages in plant height and crown width, *L*. *rupicola* had a well-developed root system, *L. ferdinandi* had thick stems but shallow roots, and *E*. *phellomanus* had the smallest crown width.

**Table 2:** Morphological characteristics of six shrub species.

Species	Plant height (cm)	Basal diameter (mm)	Root length (cm)	Crown width north-south (cm)	Crown width east-west (cm)
*C. multiflorus*	94.23 ± 4.27 b	21.2 ± 2.38 abc	31.03 ± 1.81 bc	70.00 ± 8.14 a	68.00 ± 4.93 a
*P. pedunculata*	82.27 ± 7.29 b	16.93 ± 0.79 bc	34.43 ± 1.53 abc	71.67 ± 4.48 a	68.00 ± 1.00 a
*L. ferdinandi*	87.27 ± 4.02 b	27.47 ± 4.18 a	26.23 ± 2.08 c	76.33 ± 10.9 a	57.33 ± 6.98 ab
*L. rupicola*	85.93 ± 12.43 b	19.69 ± 0.40 bc	42.17 ± 0.73 a	70.63 ± 12.06 a	41.97 ± 8.62 b
*C. arborescens*	205.17 ± 4.89 a	22.94 ± 2.16 ab	33.1 ± 2.82 abc	73.87 ± 16.34 a	68.1 ± 12.19 a
*E. phellomanus*	96.63 ± 7.27 b	14.60 ± 0.15 c	38.77 ± 5.22 ab	47.40 ± 1.15 b	37.60 ± 3.58 b

Values are presented as mean ± standard error. Different lowercase letters within a column indicate significant differences among species (p< 0.05). The same convention applies to the following tables.

Significant interspecific differences were detected in leaf functional traits and biomass accumulation among the six shrub species (p< 0.05; **Table 3**). *C*. *arborescens* demonstrated superior performance, exhibiting significantly greater values in leaf dry weight (0.04 kg), specific leaf area (SLA; 12.22 m²·kg^-^¹), leaf area per plant (0.53 m²), and leaf area index (LAI; 2.49) compared to the other species. These traits suggest that *C*. *arborescens* possesses larger, thinner leaves and a denser canopy. Consistently, this species also achieved the highest whole-plant dry weight (303.03 g) with a water content of 60.05%. In contrast, *L*. *rupicola* was characterized by the smallest leaf dry weight (0.01 kg) and leaf area per plant (0.15 m²), while *P. pedunculata* exhibited the lowest SLA (7.91 m²·kg^-^¹). *E*. *phellomanus* recorded the lowest whole-plant dry weight (69.20 g).

**Table 3:** Leaf functional traits of six shrub species.

Species	Leaf dry weight (kg)	Specific leaf area (m^2^·kg^-1^)	Leaf area per plant (m^2^)	Leaf area index	Whole plant dry weight(g) [including moisture content (Equation 1)]
*C. multiflorus*	0.03 ± 0.002 bc	8.76 ± 0.47 c	0.22 ± 0.03 b	1.01 ± 0.12 b	111.18 ± 6.62 b
					(43.91%)
*P. pedunculata*	0.03 ± 0.01 bc	7.91 ± 0.67 c	0.23 ± 0.06 b	1.02 ± 0.44 b	113.51 ± 18.04 b
					(48.95%)
*L. ferdinandi*	0.03 ± 0.01 ab	8.98 ± 0.58 c	0.31 ± 0.06 b	1.45 ± 0.23 b	153.53 ± 35.21 b
					(49.09%)
*L. rupicola*	0.01 ± 0.001 c	10.82 ± 0.55 ab	0.15 ± 0.02 b	0.87 ± 0.07 b	106.8 ± 15.16 b
					(55.14%)
*C. arborescens*	0.04 ± 0.01 a	12.22 ± 0.66 a	0.53 ± 0.07 a	2.49 ± 0.68 a	303.03 ± 102.59 a
					(60.05%)
*E. phellomanus*	0.02 ± 0.001 c	9.59 ± 2.01 bc	0.16 ± 0.02 b	1.20 ± 0.27 b	69.2 ± 7.29 b
					(60.08%)

Values are presented as mean ± standard error. Different lowercase letters within a column indicate significant differences among species (p< 0.05). Moisture content (percentage) of the whole plant dry weight is given in parentheses. The same convention applies to the following tables.

The results indicate that *C*. *arborescens* has optimal leaf traits for high light-use efficiency, coupled with superior biomass accumulation capacity and water storage, pointing to a high potential for photosynthetic productivity. Conversely, the other species displayed distinct adaptive strategies: *L*. *rupicola* likely adapts via reduced leaf area, *P*. *pedunculata* through resource conservation (indicated by low SLA), and *E*. *phellomanus* via low biomass investment. These interspecific differences reflect divergent ecological strategies shaped by evolutionary adaptation.

### 3.3 Correlation analysis of plant morphological traits

**Figure 1:**
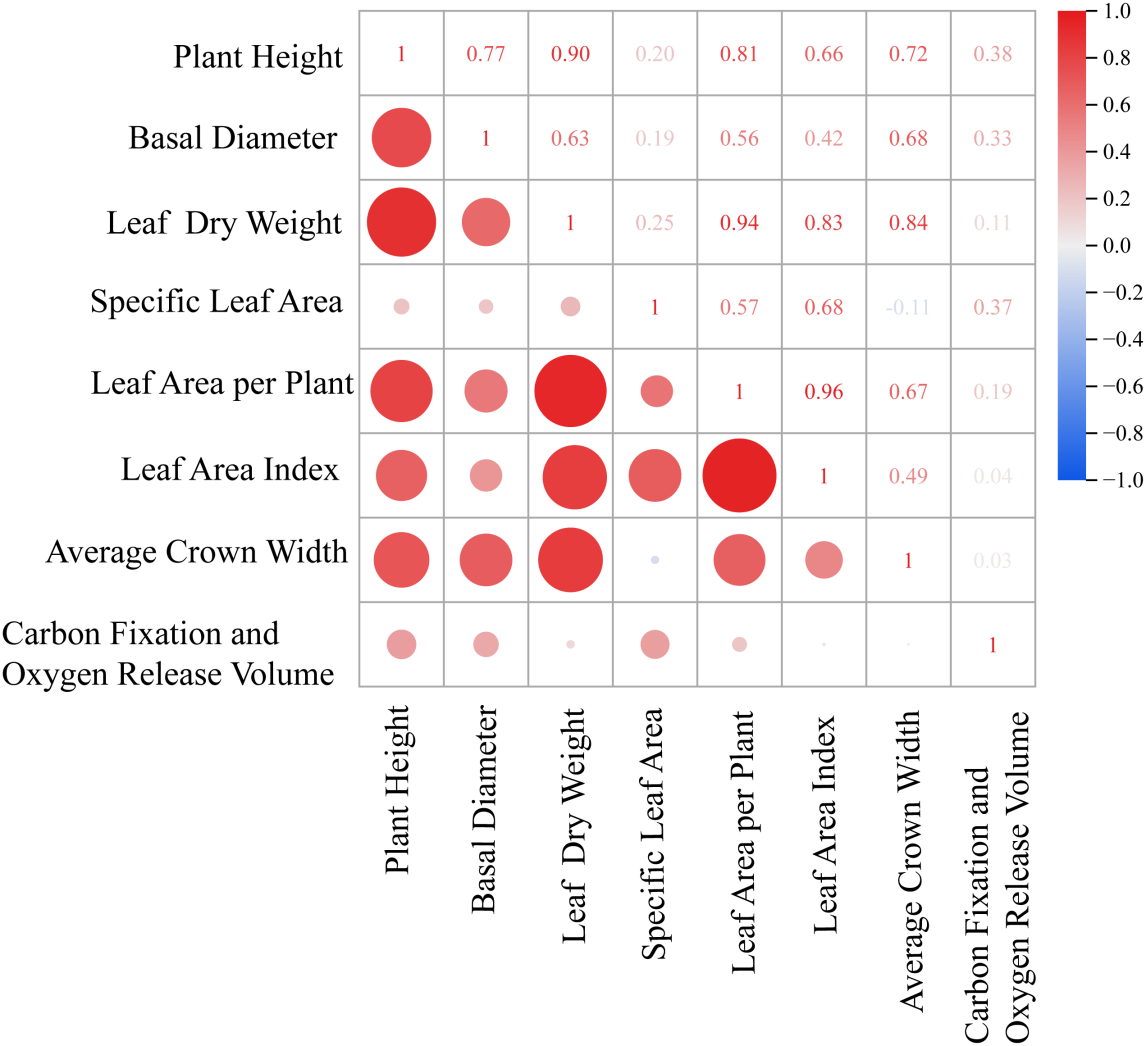
Correlation analysis chart of plant morphological parameters. The heatmap shows the Pearson correlation coefficients (R) among 11 measured traits. Color intensity and circle size indicate the strength of correlation (red: positive, blue: negative). Trait data were derived from Methods sections 2.3.1–2.3.3 (morphological traits from 30 individuals per species, whole−plant dry weight from 5 harvested individuals per species).

This study revealed the relationships among plant growth indicators through correlation analysis (**Figure 1**). Leaf dry weight (LDW) showed a strong positive correlation with leaf area per plant (LAP) (r = 0.94), and both were closely associated with leaf area index (LAI) and whole plant dry weight (WPDW), indicating a high degree of coordination between leaf biomass investment and canopy photosynthetic structure. Crown width area (CWA) was positively correlated with LAP and LAI, suggesting a coordinated relationship between horizontal canopy expansion and vertical leaf area accumulation. Plant height (PH) exhibited positive correlations with LAI, LAP, and WPDW, reflecting synchronized vertical growth with canopy development and biomass accumulation. Specific leaf area (SLA) exhibited a significant positive correlation with LAI, whereas no statistically significant relationship was detected between SLA and LDW. These results suggest that SLA is indicative of leaf construction strategy rather than direct biomass accumulation. Notably, whole plant moisture content (WPMC) was positively correlated with both SLA and LAI, suggesting that shrubs with higher tissue water content tend to develop leaves with greater area per unit mass and higher canopy leaf area. Basal diameter (BD) showed positive correlations with multiple growth traits (WPDW, LDW, LAP, CWA), supporting its role as a comprehensive indicator of overall plant growth vigor. Furthermore, the negative correlation between WPDW and WPMC implies a potential trade-off between biomass accumulation and tissue water content.

### 3.4 Photosynthetic characteristics of the six tree species

**Figure 2:**
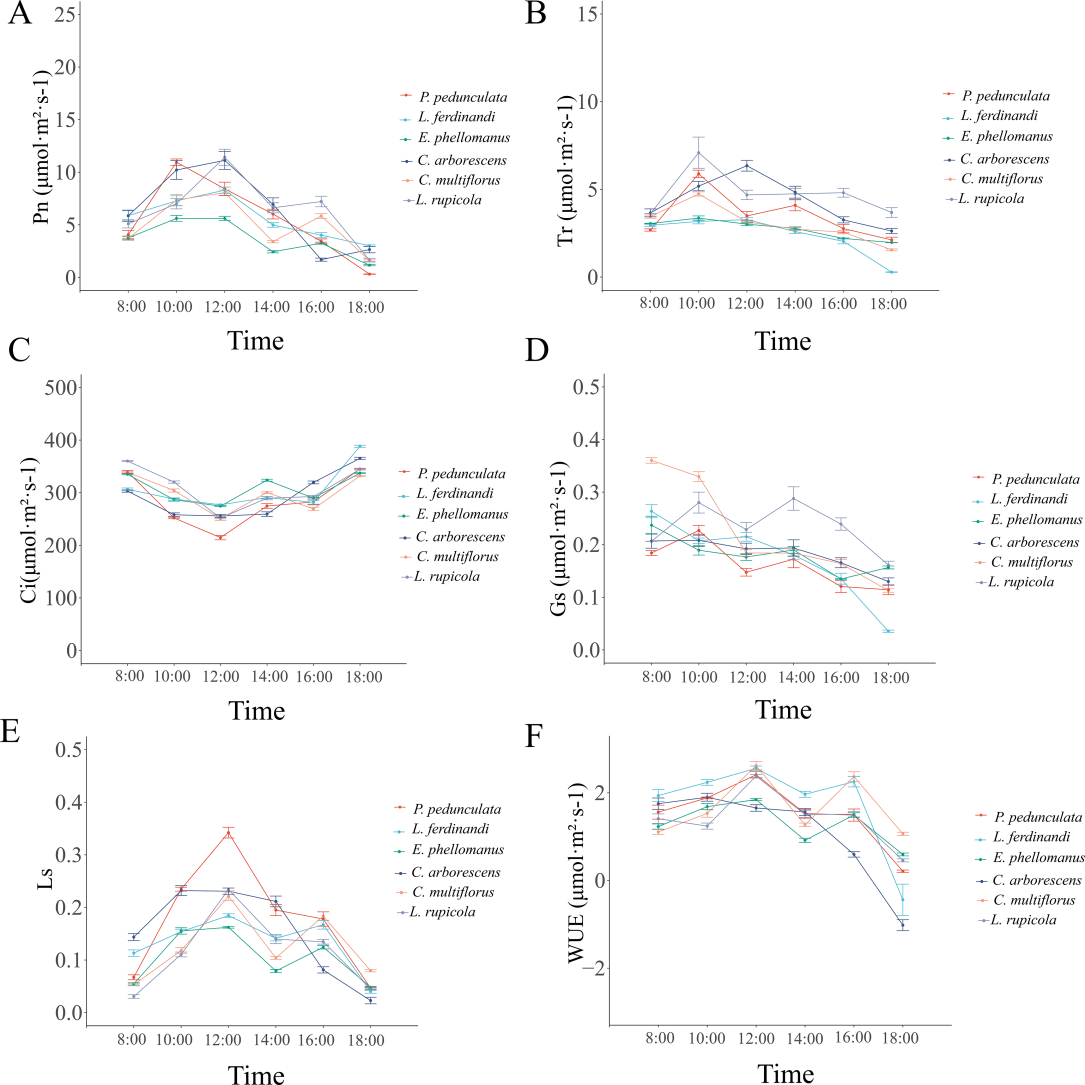
Diurnal variation patterns of photosynthetic parameters in the six shrub species. Diurnal variations in **(A)** net photosynthetic rate (Pn), **(B)** transpiration rate (Tr), **(C)** stomatal conductance (Gs), **(D)** intercellular CO_2_ concentration (Ci), **(E)** stomatal limitation value (Ls), and **(F)** water use efficiency (WUE) measured in six shrub species from 8:00 to 18:00. Measurements were conducted on clear, low−wind days between August 22 and 29, 2024, using a Li−6800 portable photosynthesis system. Three healthy, sun−exposed mature leaves per species were selected for measurement, with three technical replicates per leaf. All values are presented as means ± standard error (n=3). Pn and Tr were used to calculate WUE (WUE = Pn/Tr); Ls was derived from Ci and ambient CO_2_ concentration (Ls = 1 − Ci/Ca).

**Figure 2** illustrates the diurnal dynamics of six physiological parameters in six plant species (*C. multiflorus*, *P. pedunculata*, *L. ferdinandi*, *L. rupicola*, *C. arborescens*, and *E. phellomanus*) from 08:00 to 18:00. The net photosynthetic rate (Pn) exhibited both unimodal (*P. pedunculata*, *L. ferdinandi*, *C. arborescens*) and bimodal (*C. multiflorus*, *L. rupicola*, *E. phellomanus*) patterns, with peak values ranging from 5.61 to 11.43 μmol·m^-^²·s^-^¹. The mean daily Pn ranked in the order: *L. rupicola* > *P. pedunculata* > *C. arborescens* > *L. ferdinandi* > *C. multiflorus* > *E. phellomanus*.

Transpiration rate (Tr) also displayed unimodal and bimodal variations. *L. rupicola* showed a bimodal pattern with the highest peak at 10:00 (7.09 mmol·m^-^²·s^-^¹), while *L. ferdinandi* exhibited a unimodal peak at 12:00 (3.27 mmol·m^-^²·s^-^¹). Stomatal conductance (Gs) followed either bimodal or gradual declining patterns. *C. arborescens* displayed a typical bimodal trend, peaking at 10:00 and 16:00 (0.280 and 0.194 mol·m^-^²·s^-^¹, respectively), whereas *C. multiflorus* and *E. phellomanus* showed a gradual decline. The mean daily Gs was highest in *L. rupicola* and lowest in *P. pedunculata*.

Intercellular CO_2_ concentration (Ci) showed a “V-shaped” diurnal trend, with all species reaching a midday trough between 302.71 and 329.10 μmol·mol^-^¹. The mean daily Ci was highest in *L. rupicola* and lowest in *P. pedunculata*.

Stomatal limitation (Ls) and water use efficiency (WUE) also varied in their diurnal patterns. The mean daily Ls was highest in *P. pedunculata* and lowest in *E. phellomanus*, while the mean daily WUE was highest in *L. ferdinandi* and lowest in *C. arborescens*.

These results indicate that different shrub species exhibit distinct temporal dynamics and physiological strategies in photosynthesis, transpiration, and water use, reflecting diverse mechanisms of physiological adaptation to arid environments.

### 3.5 Photosynthetic parameters in response to environmental factors

**Figure 3:**
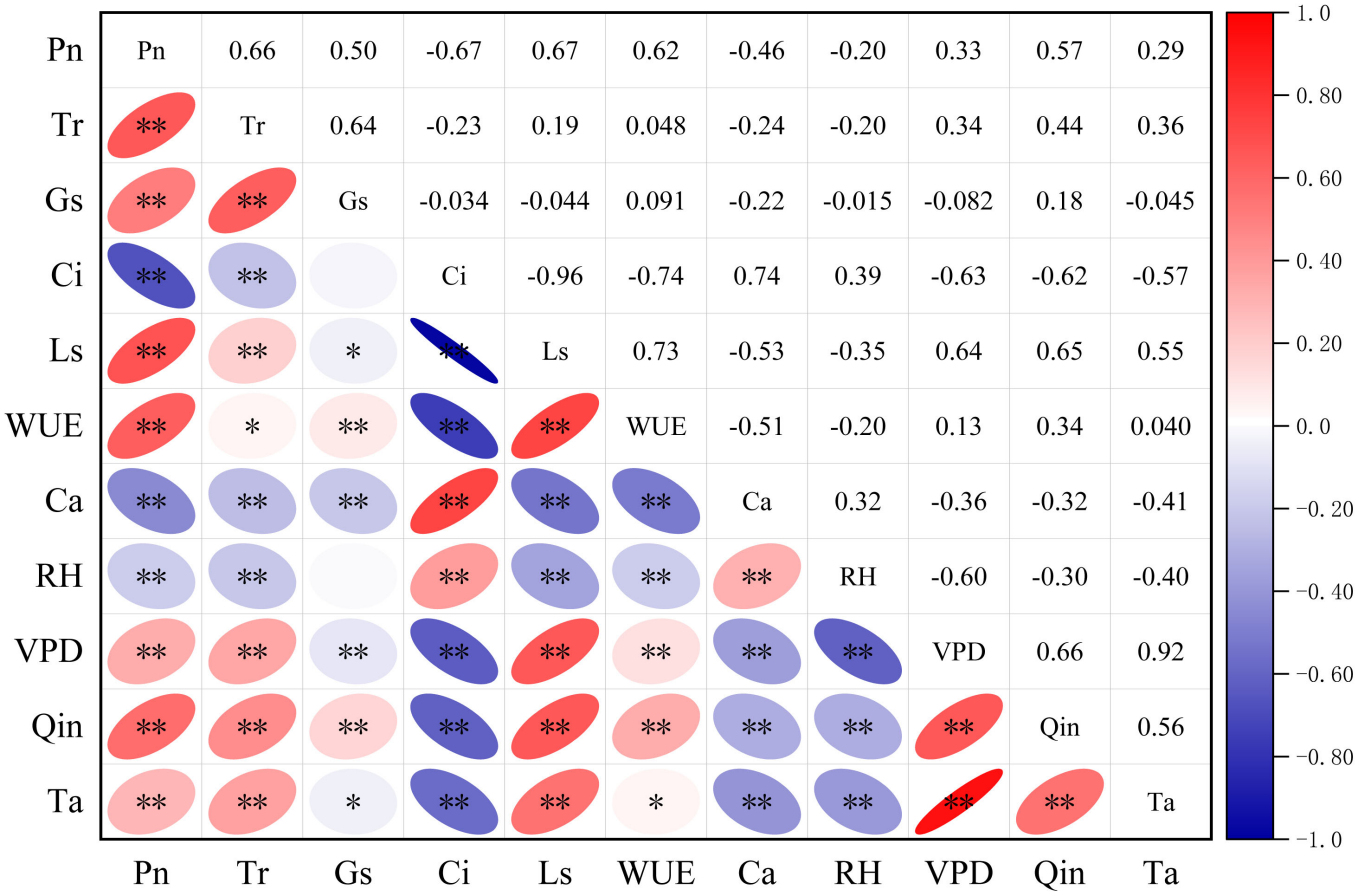
Correlation matrix between photosynthetic parameters and environmental factors. The heatmap presents the Pearson correlation coefficients among photosynthetic physiological parameters and environmental factors in six shrub species. Photosynthetic parameters include net photosynthetic rate (Pn), transpiration rate (Tr), stomatal conductance (Gs), intercellular CO_2_ concentration (Ci), stomatal limitation value (Ls), and water−use efficiency (WUE). Environmental factors consist of atmospheric CO_2_ concentration (Ca), relative humidity (RH), vapor−pressure deficit (VPD), photosynthetically active radiation (Qin), and air temperature (Ta). Data were collected under clear, low−wind conditions between August 22 and 29, 2024, using a Li−6800 portable photosynthesis system. Asterisks indicate significance levels (*p< 0.05; **p< 0.01). Color intensity and circle size reflect the strength of correlation (red: positive; blue: negative). Diagonal elements represent self−correlation (all equal to 1, not marked).

The correlation analysis revealed complex yet significant relationships among photosynthetic parameters and environmental factors (**Figure 3**). Photosynthetic rate (Pn) correlated strongly with transpiration rate (Tr). Furthermore, stomatal conductance (Gs) was positively correlated with both Pn and Tr. In contrast, intercellular CO_2_ concentration (Ci) showed a significant negative correlation with both Pn and the stomatal limitation value (Ls), suggesting that elevated Ci is associated with reduced CO_2_ utilization efficiency under stomatal limitations. Water use efficiency (WUE) was strongly positively correlated with Pn but showed no significant relationship with Tr. Regarding environmental drivers, photosynthetically active radiation (Qin) was positively correlated with Pn and Tr. Air temperature (Ta) and vapor pressure deficit (VPD) were strongly inter-correlated and both exhibited a positive association with Pn. Relative humidity (RH) showed mainly negative correlations with other variables, with its negative correlation with VPD being particularly strong. Overall, the strength of associations among physiological indicators was greater than that between physiological indicators and environmental factors, and Ci and Ls emerged as key intermediary variables linking physiological processes with environmental responses.

### 3.6 Principal component analysis on photosynthetic parameters and environmental factors

**Figure 4:**
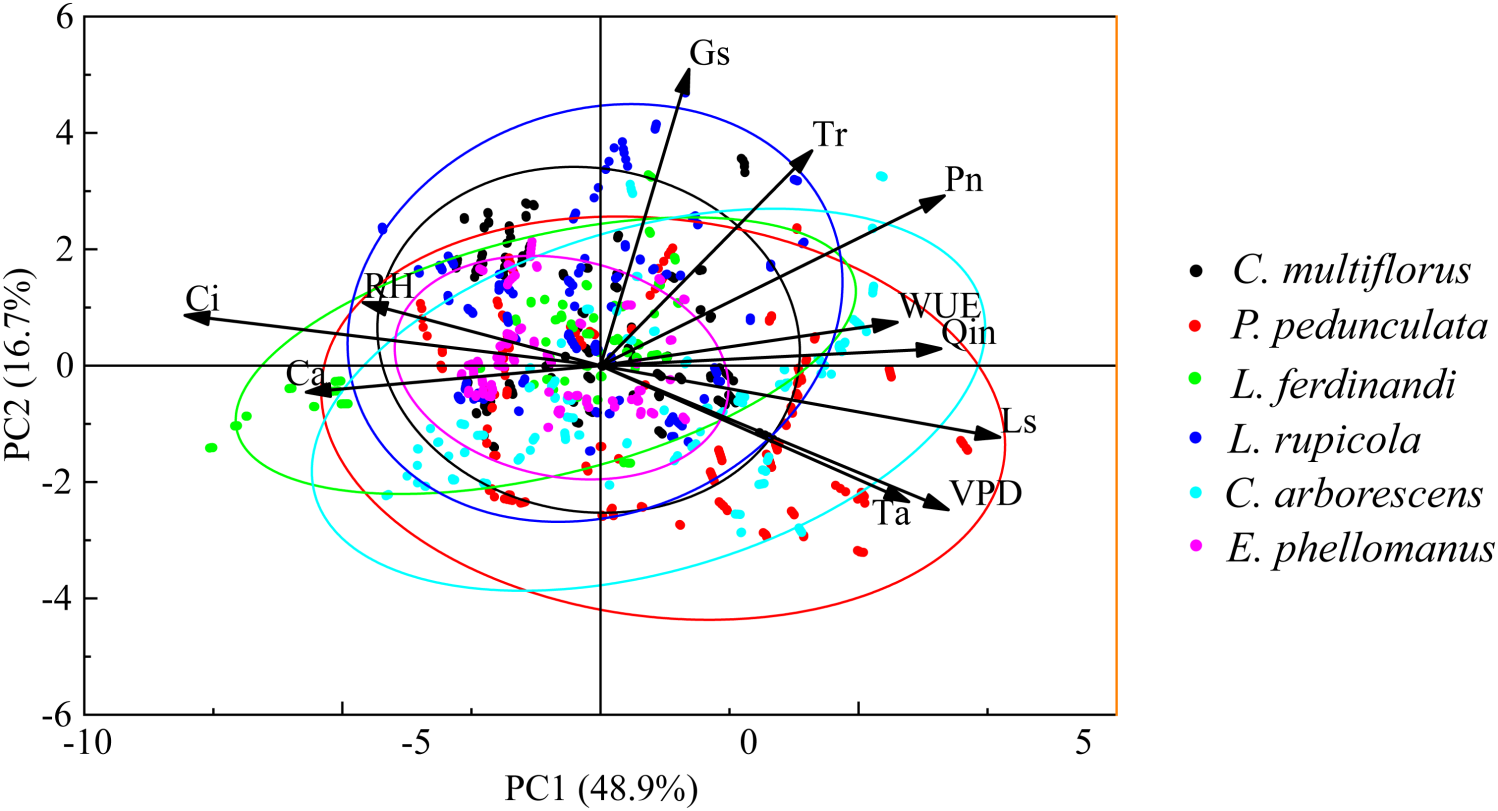
Principal component analysis (PCA) of photosynthetic parameters and environmental factors for the six shrub species. PCA was performed on photosynthetic physiological parameters (Pn, Tr, Gs, Ci, Ls, WUE) and environmental factors (Ca, RH, VPD, Qin, Ta) based on measurements taken from 08:00 to 18:00 h daily (270 observations per species, total sample size 1620). Arrows indicate the loading direction and strength of each variable in the two−dimensional principal−component space; ellipses represent the 95% confidence intervals of the observations for each species. The first principal component (PC1) explains 48.9% of the variance, and the second principal component (PC2) explains 16.7%. Spatial separation among species reflects differential responses of their photosynthetic physiological traits to environmental factors.

Principal component analysis (PCA) results showed (**Figure 4**) that the first two principal components (PC1 and PC2) collectively explained 65.6% of the total variation (PC1 accounted for 48.9%, PC2 for 16.7%). Species exhibited distinct patterns in the two-dimensional ordination space: *P. pedunculata* and *C. arborescens* were primarily distributed on the right side of the PC1 axis, showing positive correlations with photosynthetic rate (Pn), transpiration rate (Tr), stomatal conductance (Gs), and environmental factors (Qin, Ta, VPD); *L. ferdinandi* was positioned toward the negative direction of the PC2 axis, with stronger associations with intercellular CO_2_ concentration (Ci) and atmospheric CO_2_ concentration (Ca); *C. multiflorus*, *L. rupicola*, and *E. phellomanus* clustered near the center of the ordination plot and displayed weaker correlations with most indicators. Among the physiological parameters, Pn, Tr, Gs, and Ls were significantly positively correlated with the positive direction of PC1, whereas Ci and RH were negatively associated with PC1, indicating that PC1 primarily represents an opposing gradient between “photosynthesis–transpiration–stomatal behavior” and “carbon concentration–humidity.” PC2 mainly differentiated species associated with Ci and Ca, such as *L. ferdinandi*. Overall, the differences in physiological and ecological traits among species could be effectively distinguished through the associations between principal component axes and indicators, with photosynthetic and transpiration-related parameters serving as key drivers of species differentiation.

### 3.7 Comprehensive analysis of leaf-scale carbon assimilation potential and whole-plant carbon storage

#### 3.7.1 Estimation and significance of leaf-scale carbon assimilation potential

**Figure 5:**
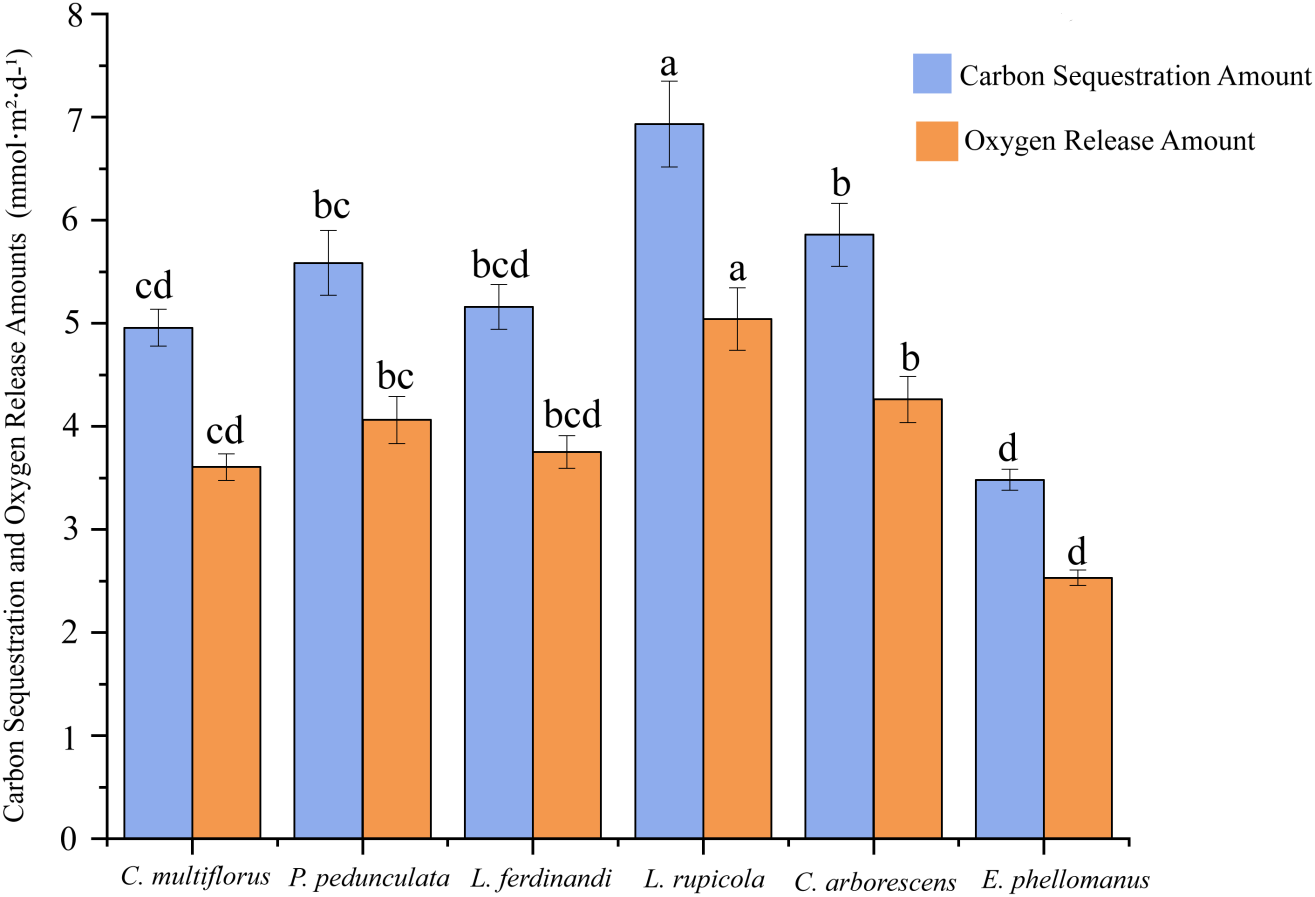
Carbon sequestration and oxygen release capacities of six shrubs species. The daily carbon sequestration (CO_2_ fixation) and oxygen release (O_2_ production) per unit leaf area were estimated based on the integration of diurnal net photosynthetic rate (Pn) curves. Values were converted using Equations 7 and 8, respectively, with daytime net assimilation adjusted for nocturnal dark respiration (accounting for 20% of daily assimilation). Data are presented as mean ± standard error (n=3). Different lowercase letters indicate significant differences among species (p< 0.05, one−way ANOVA followed by Duncan’s multiple range test).

Based on the integration of the diurnal net photosynthetic rate (Pn) curves, we estimated the daily carbon sequestration and oxygen release per unit leaf area for the six shrub species (**Figure 5**). This estimate directly reflects the cumulative carbon assimilation capacity of leaves over a full day and serves as a core physiological indicator of plant photosynthetic production potential. The results showed significant interspecific differences in the estimated daily carbon sequestration and oxygen release among the six shrubs: *L. rupicola* had the highest value (6.93 g·m^-2^·d^-1^), followed by *C. arborescens* (5.86 g·m^-2^·d^-1^) and *P. pedunculata* (5.59 g·m^-2^·d^-1^). *L. ferdinandi* and *C. multiflorus* exhibited similar, moderate values, while *E. phellomanus* had the lowest estimate (3.48 g·m^-2^·d^-1^).

Mechanistically, the significantly higher daily carbon sequestration and oxygen release of *L. rupicola* and *C. arborescens* can be attributed to their superior diurnal net photosynthetic performance, including both higher instantaneous photosynthetic peaks and longer durations of efficient photosynthesis throughout the day, which collectively led to greater cumulative carbon assimilation. It should be noted that this estimation focuses on the photosynthetic accumulation effect at the leaf level. Its primary value lies in clearly revealing the inherent differences among shrub species in carbon acquisition efficiency per unit leaf area, thereby providing key physiological evidence for further evaluating species’ photosynthetic production capacity and ecological service potential.

#### 3.7.2 Whole-plant carbon storage characteristics based on measured biomass and carbon content

To more realistically assess plant carbon storage capacity, this study calculated the whole-plant carbon storage of individual shrubs for all six species (**Table 4**) through destructive whole-plant harvesting to obtain dry biomass, combined with carbon content measurements of leaves, stems, and roots. Significant differences were observed among the six shrubs in terms of biomass accumulation, allocation patterns, and carbon storage characteristics. Based on biomass allocation ratios and carbon content per unit biomass (carbon storage efficiency), the species can be preliminarily categorized into three types:

**Table 4:** Biomass allocation and organ carbon content of the six shrub species.

Species	Biomass allocation (%)			Organ carbon storage (g/plant)			Total carbon storage (g/plant)	C Content per biomass (g C/100g DW)
	Leaf	Stem	Root	Leaf carbon storage (g/plant)	Stem carbon storage (g/plant)	Root carbon storage (g/plant)		
*C. multiflorus*	22.42±2.62 a	43.53± 4.81 b	34.0± 7.12 ab	9.95± 2.17 bcd	19.25± 2.4 b	14.61± 2.9 a	43.81± 4.53 b	39.40± 0.59 bc
*P. pedunculata*	24.50±3.54 a	44.84± 5.87 b	30.66± 5.27 ab	12.26± 4.47 abc	21.92± 4.05 b	15.48± 5.6 a	49.66± 13.65 b	43.74± 0.54 a
*L. ferdinandi*	25.31 ± 12.8 a	39.80± 8.27 b	34.87± 4.56 a	13.32± 4.06 ab	24.82± 15.54 b	20.96± 12.35 a	59.09± 25.52 b	38.17± 1.21 c
*L. rupicola*	13.66 ± 4.01 a	56.61± 1.24 a	29.72± 3.04 ab	6.09± 0.98 d	26.22± 6.5 b	12.82± 3.87 a	45.13± 9.87 b	42.49± 1.81 ab
*C. arborescens*	16.24 ± 5.19 a	59.84± 6.61 a	23.92± 6.44 b	16.54± 4.25 a	76.34± 48.54 a	27.99± 18.1 a	120.86 ± 70 a	40.01± 0.67 bc
*E.phellomanus*	24.39 ± 2.54 a	37.93± 1.65 b	37.68± 3.72 a	7.02± 0.55 cd	11.56± 1.75 b	10.9± 2.52 a	29.49± 4.41 b	42.85± 3.92 ab

Values are mean ± standard error. Different lowercase letters within a column indicate significant differences among species (p< 0.05).

*C. arborescens* is classified as a “high-accumulation, stem-dominated” type. It exhibited the highest whole-plant dry weight (303.03 g) and total carbon storage per plant (120.86 g), with carbon primarily stored in stems (accounting for 63.2%), indicating prominent carbon sequestration potential.

*C. multiflorus*, *L. ferdinandi*, *P. pedunculata*, and *L. rupicola* are categorized as “balanced-investment” types. Among them, *P. pedunculata* and *L. rupicola* showed higher carbon content per unit biomass (43.74 g C/100g DW and 42.49 g C/100g DW, respectively), reflecting more efficient carbon storage. In contrast, *C. multiflorus* and *L. ferdinandi* displayed typical balanced biomass allocation (with similar investment proportions across organs) but relatively lower unit carbon content (39.40 g C/100g DW and 38.17 g C/100g DW, respectively).

*E. phellomanus* is characterized as a “conservative, belowground-investment” type. It allocated the highest proportion of biomass to roots (37.68%); however, both its total biomass and carbon storage were low, indicating relatively limited carbon sequestration contribution.

### 3.8 Cluster analysis based on leaf carbon sequestration and oxygen release

**Figure 6:**
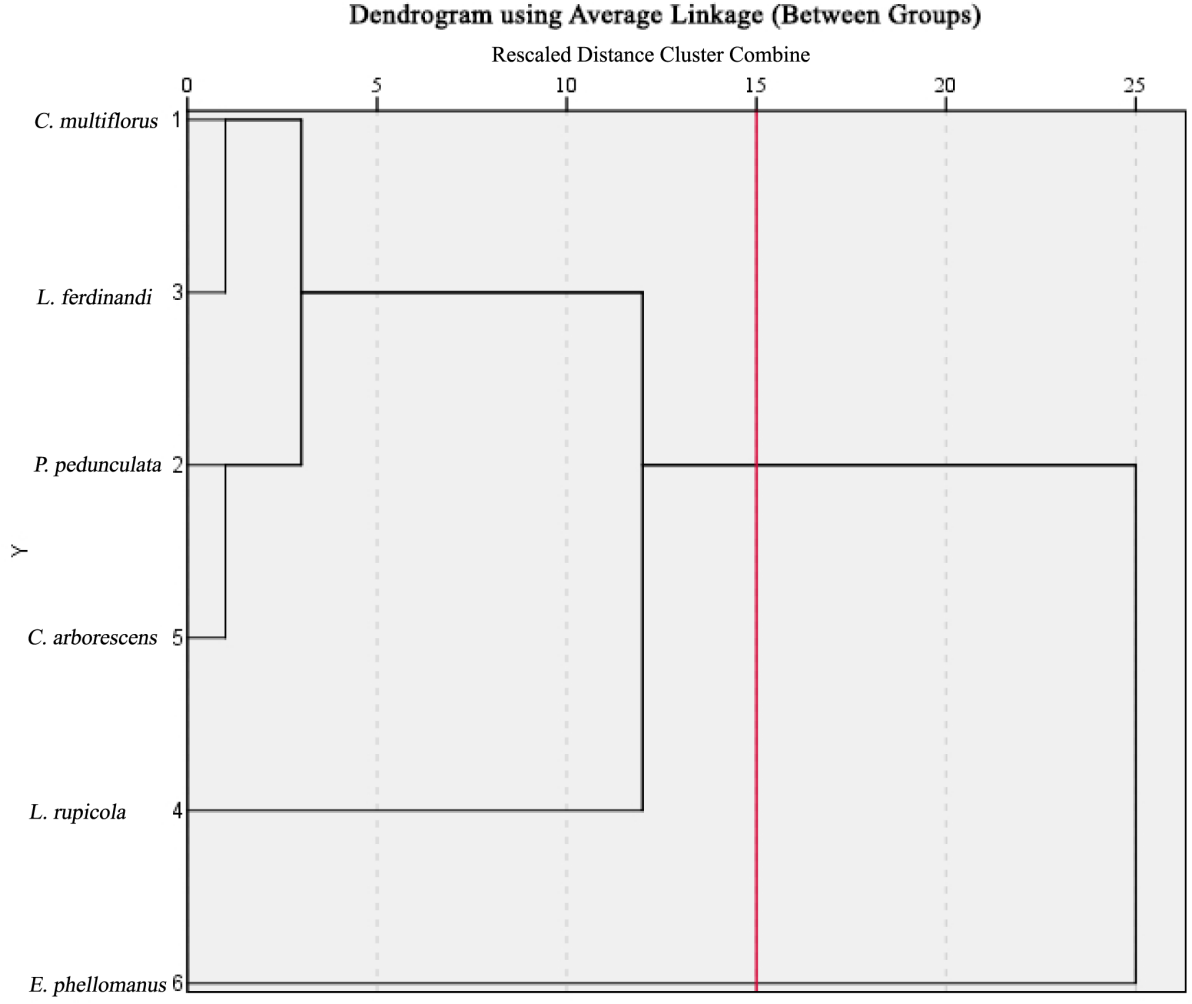
Cluster analysis based on leaf carbon sequestration and oxygen release capacities. Cluster analysis was performed using the unweighted pair group method with arithmetic mean (UPGMA) based on carbon sequestration and oxygen release per unit leaf area of six shrub species. The distance scale (0–25) indicates the standardized distance between clusters. The results show that the six species can be divided into two main groups: Group I includes *C. multiflorus*, *L. ferdinandi*, *P. pedunculata*, *C. arborescens* and *L. rupicola*; Group II contains only *E. phellomanus*. The clustering outcome reflects, from a statistical perspective, functional differentiation in leaf carbon acquisition efficiency among the shrub species.

To investigate the similarity and grouping characteristics of leaf carbon assimilation function among the six shrub species, their carbon sequestration and oxygen release data were standardized and subjected to hierarchical cluster analysis (**Figure 6**). When the Euclidean distance was set to 15, the tested shrubs could be divided into two clusters: *C. arborescens* was grouped alone into one cluster, demonstrating relatively strong carbon sequestration and oxygen release capacity, while *C. multiflorus*, *L. ferdinandi*, *P. pedunculata*, *L. rupicola*, and *E. phellomanus* were grouped into the other cluster, showing relatively weaker carbon sequestration and oxygen release capacity. This result further confirms, from a statistical classification perspective, the functional differentiation among different shrubs in terms of leaf carbon acquisition efficiency.

### 3.9 Correlation between leaf carbon assimilation potential and key functional traits

**Figure 7:**
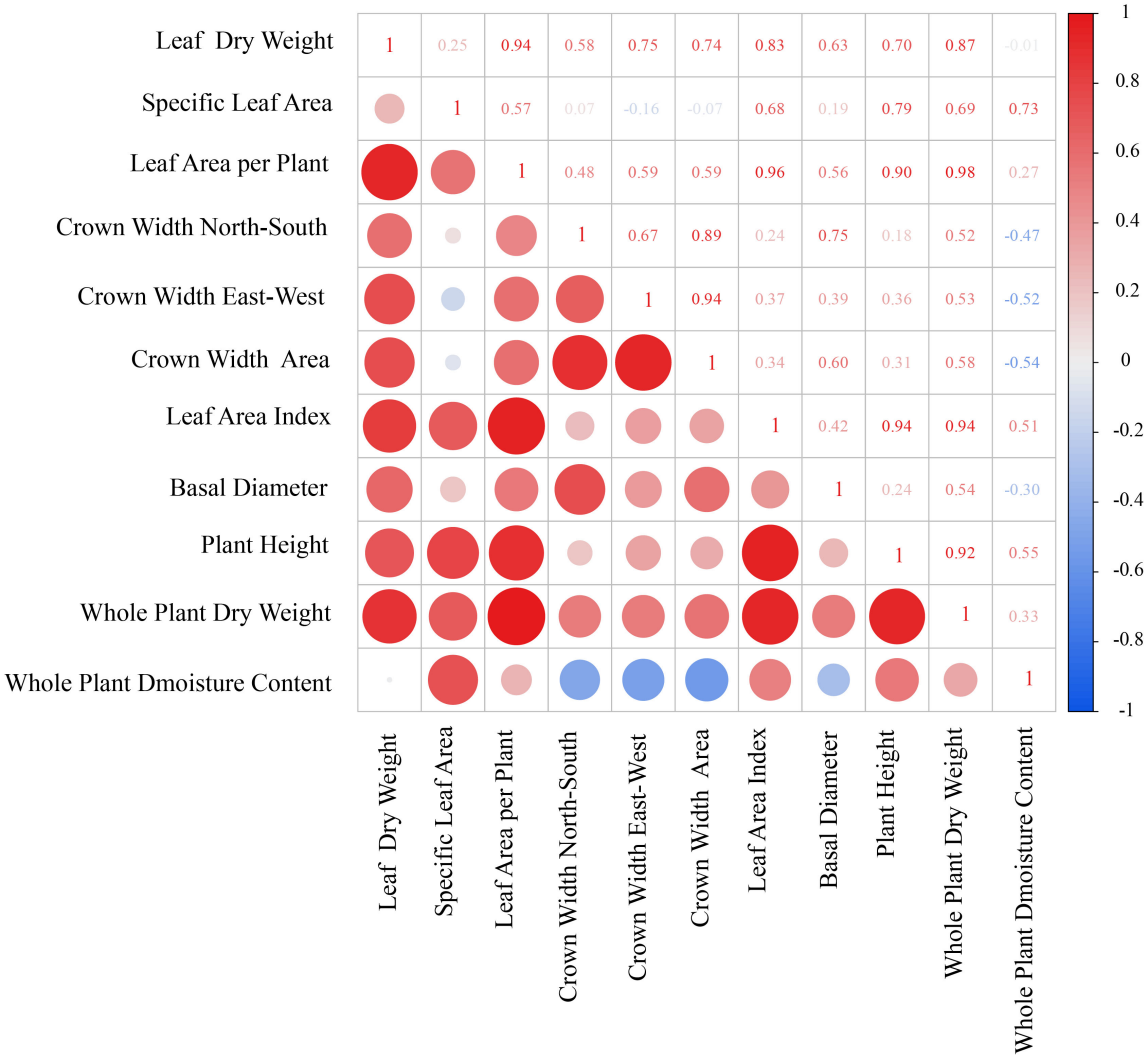
Correlation matrix between carbon fixation and oxygen release and shape. The figure shows the Pearson correlation coefficient matrix between morphological indices (plant height, basal diameter, leaf dry weight, specific leaf area, leaf area per plant, leaf area index, average crown width) and carbon fixation & oxygen release per unit leaf area across six shrub species. Data were derived from measurements described in Methods sections 2.3.1–2.3.6. Numerical values indicate correlation coefficients; color intensity and circle size reflect the strength of correlation (red: positive, blue: negative).

To systematically analyze the intrinsic mechanisms driving differences in carbon fixation and oxygen release capacity (CFORV) at the leaf scale and clarify its relationship with overall morphological structure, we conducted a correlation analysis (**Figure 7**). The results showed that the estimated leaf carbon assimilation potential (CFORV) did not exhibit significant correlations with any of the measured overall morphological indicators, including plant height (PH), basal diameter (BD), leaf dry weight (LDW), leaf area per plant (LAA), leaf area index (LAI), and average crown width (ACW). This key finding indicates that leaf-level carbon assimilation potential is a trait dimension relatively independent of plant morphological construction scale. At the same time, systematic synergistic relationships existed among the morphological traits (**Figure 7**): leaf dry weight (LDW) showed a significant positive correlation with leaf area per plant (LAA); basal diameter (BD) exhibited a significant positive correlation with average crown width (ACW); and plant height (PH) was significantly positively correlated with both LAA and LAI. Particularly noteworthy is the significant negative correlation between specific leaf area (SLA) and ACW, which may suggest a trade-off between leaf construction strategy (thin and efficient) and canopy spatial expansion strategy.

## 4 Discussion

### 4.1 Shrub physiological response patterns driven by diurnal environmental variation

The diurnal variations in photosynthetically active radiation and air temperature observed in this study significantly drove an increase in vapor pressure deficit (VPD) and a decrease in relative humidity (RH), a pattern consistent with the general eco-physiological responses typical of arid and semi-arid regions (Tang et al., 2024). Under the conditions of potentially intensifying water stress in the afternoon, all six tested shrub species exhibited a trend of “peaking in the morning and declining significantly in the afternoon” for both net photosynthetic rate (Pn) and transpiration rate (Tr). Crucially, the coordinated decline in stomatal conductance (Gs) and the “V-shaped” fluctuation in intercellular CO_2_ concentration (Ci) jointly indicate that stomatal limitation was the core physiological mechanism driving the afternoon attenuation of photosynthesis. This aligns with the findings of Wang et al. (2023) regarding the regulation of plant photosynthetic potential by environmental stress (Wang et al., 2023b). The intensity of response to these environmental changes varied among species, revealing interspecific differentiation in water-use strategies and stress tolerance. For instance, *L. rupicola* and *C. arborescens* maintained relatively high peak photosynthetic capacity, whereas *E. phellomanus* exhibited the lowest daily carbon fixation. This differential response reflects the diverse adaptation strategies of native shrubs in arid regions, corroborating the findings of Pompelli et al. (2024), who reported that *Cenostigma pyramidale* exhibits significant physiological adaptations to cope with extreme water deficit (Pompelli et al., 2024).

### 4.2 Interspecific differences in morphological and biomass allocation strategies and their ecological significance

The response intensity of different species to the aforementioned stresses exhibits significant divergence, which is closely related to their inherent morphological structure and biomass allocation strategies. The six shrub species studied showed pronounced differences in plant height, crown width, root system architecture, and biomass allocation patterns, visually reflecting the diverse ecological adaptation strategies among species. *C. arborescens* adopts a typical high-investment, high-return strategy, demonstrating significant advantages in plant height, crown width, leaf area index (LAI), and stem biomass allocation. By establishing a photosynthetic system characterized by high LAI and a large crown, and allocating more resources to long-term carbon pools such as stems, this species maintains a high photosynthetic peak, achieving maximized whole-plant biomass and carbon storage. This reflects its strong competitive ability to rapidly occupy ecological niches and efficiently sequester carbon in relatively resource-abundant habitats (Castorena et al., 2022; Ngidi et al., 2024). These results confirm that leaf and canopy morphological traits are key structural drivers of plant productivity (Wang and Sheng, 2022).

In contrast, the well-developed root system of *L. rupicola* and the higher root biomass allocation of *E. phellomanus* represent a more conservative survival strategy. This pattern of increased investment in belowground structures helps enhance water and nutrient acquisition capabilities in barren or arid habitats, improving establishment and survival probabilities (Rossi et al., 2020; Rao et al., 2023). These significant “trade-offs” in morphology and biomass allocation are concrete manifestations of species adapting to specific environmental pressures and optimizing the allocation of limited resources (Wang et al., 2022).

### 4.3 Decoupling and association between photosynthetic carbon assimilation potential and whole-plant carbon sink function

Plant growth is essentially the process of carbon compound accumulation through CO_2_ fixation via photosynthesis. However, accurately assessing plant carbon sequestration capacity presents challenges related to scale and methodology (Boatwright et al., 2022). Previous studies have indicated that, due to significant interspecific differences in morphological traits such as leaf area index and crown size among shrubs, relying solely on per-unit-leaf-area carbon fixation cannot fully reflect their overall carbon sequestration potential. This aligns with findings in forest studies that carbon sequestration capacity is influenced by multiple factors such as stand age and site conditions (Wang et al., 2020; Ma et al., 2021). Therefore, this study moves beyond single-scale leaf-level estimation by integrating shrub morphological traits (e.g., plant height, basal diameter, biomass allocation) with leaf photosynthetic parameters to comprehensively compare carbon acquisition strategies across species.

A comparison between the daily per-unit-leaf-area carbon sequestration/oxygen release potential and the measured whole-plant carbon storage reveals a clear decoupling effect: *L. rupicola* shows the highest daily carbon fixation potential per unit leaf area, yet its total carbon storage per plant does not correspondingly rank highest. Conversely, *C. arborescens* achieves the highest whole-plant carbon storage while exhibiting only moderate per-unit-leaf-area carbon fixation efficiency. This pattern highlights the significant scale-dependent dissociation between leaf-level assimilation potential and whole-plant carbon accumulation.

The adaptive strategy of *C. arborescens* exemplifies this mechanism—its large individual size (high biomass accumulation) and stem-dominated biomass allocation effectively compensate for the relatively lower per-unit-leaf-area carbon fixation efficiency, ultimately maximizing whole-plant carbon storage. This result resonates with the cross-biome study by Cabon et al. (2022) published in *Science*, which emphasized that the decoupling between carbon assimilation (physiological potential) and growth (carbon sink capacity) is a widespread ecological process. Whole-plant carbon sink capacity is essentially the integrated outcome of physiological traits and morphological structure, which clearly explains why “high photosynthetic efficiency does not necessarily translate into high carbon storage”.

This decoupling phenomenon has direct implications for species selection in ecological restoration. If the goal is to achieve rapid aboveground carbon accumulation, species with strong morphological construction capabilities like *C. arborescens* should be prioritized (Lv et al., 2023). If the aim is to achieve stable and sustained carbon fixation under harsh conditions, species with high per-unit-leaf-area efficiency and strong stress resistance, such as *L. rupicola*, may be more suitable.

It is important to note that the method used in this study to estimate whole-plant carbon sequestration based on the integration of instantaneous photosynthetic rates over a day has limitations. The sparse canopy structure and high spatial heterogeneity of leaves in arid shrubs further amplify the estimation error when scaling from single-leaf measurements to the whole canopy. Therefore, the “carbon assimilation potential” calculated from peak-growing-season photosynthetic parameters in this study should be strictly interpreted as “a relative comparison of per-unit-leaf-area carbon assimilation potential among different species under measured daytime conditions” and should not be equated directly with a species’actual ecosystem carbon sink contribution. Scientifically reliable carbon sink assessment requires destructive whole-plant biomass measurement (Huera-Lucero et al., 2024) or long-term ecosystem flux monitoring (Sun et al., 2024). By simultaneously presenting leaf-scale potential estimates and whole-plant measured data, this study not only clearly reveals the scale-decoupling mechanism between carbon assimilation and carbon storage in arid shrubs but also provides a more rigorous and comprehensive research framework for accurate carbon sink assessment of shrubs in this region.

Notably, as dominant components of arid ecosystems, the scale decoupling observed in shrub carbon sink functions suggests that regional carbon cycle models need to incorporate species-specific carbon allocation strategies and morphology-physiology coordination mechanisms to avoid estimation biases arising from reliance on single leaf-scale parameters. This is crucial for improving the accuracy of carbon sink assessments in arid ecosystems and supporting regional carbon neutrality goals.

### 4.4 Quantitative identification of key trait associations and ecological strategies

Correlation analysis revealed the morphology–physiology coordination patterns and strategy differentiation among the six shrub species. Morphological correlation analysis showed that crown width exhibited a strong positive correlation with leaf area indicators (leaf area per plant, leaf area index), indicating that horizontal canopy expansion and vertical leaf area accumulation form a coordinated light-capturing module to maximize light-use efficiency (Patiño et al., 2012). In contrast, whole-plant water content was significantly negatively correlated with crown width, suggesting a potential trade-off in resource allocation between rapid canopy expansion for light acquisition and water conservation. Such trade-offs reflect how plants optimize the balance between growth and water management under limited resources (Piña et al., 2024). Rapid canopy expansion typically requires substantial investment of carbon and water, which may limit resources available for structural biomass accumulation or water storage. For instance, under water stress, plants may adjust their hydraulic traits and non-structural carbohydrate responses to maintain water balance (Wang et al., 2024b). The strategic choice between “rapid expansion to compete for light” and “robust growth to maintain water balance” is crucial for shrub survival in arid or resource-limited environments.

Principal component analysis (PCA) further quantified the positions of these shrubs in the physiological-ecological strategy space. PCA is a commonly used dimensionality-reduction technique capable of revealing underlying structures in high-dimensional data (Nguyen et al., 2021a). In this study, PC1 (48.9%) represented a strategy axis characterized by the synergy between “high photosynthesis-transpiration-stomatal conductance” and high light-temperature conditions. Species clustered toward the positive direction of PC1 (*P. pedunculata* and *C. arborescens*) exhibited typical high-resource-acquisition traits, indicating that these species tend to achieve rapid growth and biomass accumulation through high photosynthetic and transpiration rates in relatively resource-rich environments (Liu et al., 2024). The negative direction of PC1 was associated with high intercellular CO_2_ concentration and high humidity, suggesting alternative carbon-use strategies. PC2 (16.7%) primarily differentiated *L. ferdinandi*, which was closely related to high atmospheric and intercellular CO_2_ concentrations, implying a specialized carbon-use strategy. The PCA results constructed a continuous spectrum of species differentiation along a resource-use gradient from a multivariate perspective. Cluster analysis based on leaf carbon assimilation potential corroborated the PCA findings: *C. arborescens* was grouped separately due to its higher carbon assimilation potential, consistent with its classification as a high-resource-acquisition type in the PCA.

In summary, arid-zone shrubs form a continuous strategy spectrum from high resource acquisition to specialized adaptation through modular coordination of morphological traits and differentiated integration of physiological traits. This provides a functional-trait basis for species selection and ecological restoration (Pierce et al., 2022).

### 4.5 Implications for vegetation restoration and carbon sink forestry construction in the arid zone of the Qilian Mountains

Based on the aforementioned findings, species selection for carbon-oriented vegetation restoration in the arid zone of the Qilian Mountains should follow the following principles. First, clear objectives and matching indicators are crucial. If the restoration project aims to achieve rapid above-ground biomass accumulation in the short term, for example in relatively resource-abundant environments, priority should be given to species such as *C. arborescens*, which possess tall plant stature and efficient biomass allocation structures. These species exhibit a “high-investment-high-return” growth strategy, achieving higher light capture and carbon assimilation through larger photosynthetic areas and denser canopies, making their whole-plant carbon storage metrics particularly valuable (Xu et al., 2024).

Second, leaf-scale indicators should be used with caution in species selection. Species with high leaf-scale carbon assimilation potential (e.g., *L. rupicola*) may indicate strong photosynthetic stress resistance and high water-use efficiency, making them more suitable as pioneer or companion species in arid and poor habitats (Hu et al., 2023). However, their ultimate carbon accumulation may be limited by growth scale. Therefore, their overall carbon sink contribution should not be evaluated based solely on leaf-scale efficiency; rather, this trait should be viewed as a manifestation of physiological resilience under extreme environments.

Third, a multidimensional comprehensive evaluation system should be adopted. An ideal carbon-sink species should combine high leaf photosynthetic efficiency (intrinsic potential), superior morphological construction capacity (structural foundation for translating physiological potential into large-scale carbon accumulation), and strong environmental adaptability (Wang et al., 2024c). It is recommended to establish a multi-indicator evaluation framework that includes leaf physiological parameters (e.g., photosynthetic rate, water-use efficiency), individual morphological traits (e.g., plant height, crown width, basal diameter), biomass allocation patterns (e.g., root-shoot ratio, biomass proportion of different organs), and stress-tolerance indicators.

Furthermore, given the pronounced seasonal dynamics of plant carbon sequestration capacity, future research should conduct continuous monitoring throughout the entire growing season to obtain more accurate annual carbon sink assessments. For instance, the carbon balance (including both carbon uptake and release) at the annual scale is essential for accurately reflecting the net carbon sink function of an ecosystem (Deng et al., 2024). Continuous monitoring will provide a solid foundation for the sustainable management and enhancement of carbon sink functions in arid shrub ecosystems.

## Data Availability

The original contributions presented in the study are included in the article/supplementary material. Further inquiries can be directed to the corresponding author/s.
